# Alterations of erythrocyte rheology and cellular susceptibility in end stage renal disease: Effects of peritoneal dialysis

**DOI:** 10.1371/journal.pone.0171371

**Published:** 2017-02-03

**Authors:** Nesrin Zeynep Ertan, Semra Bozfakioglu, Elif Ugurel, Mukaddes Sinan, Ozlem Yalcin

**Affiliations:** 1 Department of Physiology, Istanbul University, Istanbul, Turkey; 2 Department of Nephrology, Istanbul University, Istanbul, Turkey; 3 Department of Physiology, Koc University, Istanbul, Turkey; Université Claude Bernard Lyon 1, FRANCE

## Abstract

In this study, we investigated the effects of peritoneal dialysis on hemorheological and hematological parameters and their relations with oxidant and antioxidant status of uremic patients. Hemorheological parameters (erythrocyte deformability, erythrocyte aggregation, osmotic deformability, blood and plasma viscosity) were measured in patients with renal insufficiency undergoing peritoneal dialysis (PD) and volunteers. Erythrocyte deformability, osmotic deformability and aggregation in both autologous plasma and 3% dextran 70 were measured by laser diffraction ektacytometry. Enzyme activities of glutathione peroxidase, superoxide dismutase and catalase were studied in erythrocytes; lipid peroxidation was studied by measuring the amount of malondialdehyde in both erythrocytes and plasma samples. Blood viscosity at native hematocrit was significantly lower in PD patients at all measured shear rates compared to controls, but it was high in PD patients at corrected (45%) hematocrit. Erythrocyte deformability did not show any difference between the two groups. Osmotic deformability was significantly lower in PD patients compared to controls. Aggregation index values were significantly high in PD patients in plasma Catalase and glutathione peroxidase activities in erythrocytes were decreased in PD patients whereas superoxide dismutase activity was increased compared to controls. Malondialdehyde was significantly increased in erythrocytes and plasma samples of PD patients which also shows correlations with aggregation parameters. It has been concluded that erythrocytes in PD patients are more prone to aggregation and this tendency could be influenced by lipid peroxidation activity in patient’s plasma. These results imply that uremic conditions, loss of plasma proteins and an increased risk of oxidative stress because of decreasing levels of antioxidant enzymes affect erythrocyte rheology during peritoneal dialysis. This level of distortion may have crucial effects, impairing the blood flow dynamics and causing inadequate microcirculatory perfusion.

## Introduction

Erythrocytes are continuously exposed to hyperosmolarity in the kidney medulla. While they are passing through these areas, the contact time of erythrocytes is too short to induce eryptosis [1, 2]. Erythrocytes normally defend themselves via their antioxidant systems against oxidative stress *in vivo*. They are also protected by the inhibition of cation channels by Cl^-^ and blunting sphingomyelinase by high urea concentration prevailing in kidney medulla [2]. Osmotic cell shrinkage opens non-selective cation channels in the erythrocyte membrane [3]. These channels are also activated by oxidative stress. Hence, exposure to osmotic shock or oxidative stress triggers Ca^2+^ uptake of erythrocytes and Ca^2+^ stimulates cell membrane scrambling with breakdown of phosphatidylserine asymmetry of the erythrocyte membrane. Thus, this leads to the translocation of phosphatidylserine to the erythrocyte surface and subsequent erythrocyte death. [2–4]. Kidney failure also causes decrement in their capacity of producing hormones, particularly erythropoietin (EPO). EPO is an essential hormone for erythrocyte production and so, anemia commonly occurs in people with end stage renal disease (ESRD) [5]. Besides the lack of EPO, shortened erythrocyte lifespan has been accepted as one of the contributory factors to anemia in patient with ESRD [6, 7].

Peritoneal dialysis (PD) is one of the most popular dialysis methods in patients with ESRD since 1980s. In that method, a soft catheter is used to fill the abdomen with a cleansing liquid. The walls of the abdominal cavity are lined with a membrane (peritoneum), which allows waste products and extra fluid to pass from blood into the dialysis solution. The solution contains dextrose that will pull wastes and extra fluid into the abdominal cavity. These wastes and fluid then leave the body when the dialysis solution is drained. In the literature, several researchers have investigated the oxidative and osmotic effects of PD on blood. One of these studies showed significant changes of antioxidant enzyme activities in PD patients [8]. They also pointed out that the PD provided better antioxidant protection than other types of therapy in ESRD. In another study, hemodialysis, peritoneal dialysis and predialysis patients had significantly higher levels of products derived from molecular oxidation with a significant decrease in antioxidant enzymes than the control, but PD group had a better oxidative balance compared to others [9].

Hemorheological studies have been performed to explain the consequences of anemia in patients with ESRD, who mostly use erythropoietin to cure anemia caused by the low production of EPO. One of these studies showed decreased erythrocyte deformability in PD patients compared to control subjects, whereas this parameter was not altered in hemodialysis patients [10]. They also reported that the activity of antioxidant enzymes did not change in both dialysis groups. In another study, uremic patients had lower erythrocyte deformability than control subjects [11]. Szikszai *et al*. showed increased relative cell transit time, which is negatively correlated with the erythrocyte deformability and malondialdehyde (MDA) levels, not only before but also after hemodialysis compared to controls [12]. These results concluded that hemodialysis (HD) did not cause any erythrocytes injury [12]. It was also observed in another study that the patients with renal insufficiency had significantly increased aggregation index (AI) and significantly reduced elongation index (EI) values. It was supposed that imbalance between catalase (CAT) and superoxide dismutase (SOD) activities might play crucial role in the impairment of erythrocyte rheological properties [13]. In our study, we focused on peritoneal dialysis and aimed to investigate its effects on the rheological parameters and the relationship with the oxidative enzymes in patients undergoing peritoneal dialysis.

## Materials and methods

### Selection criteria for patients and controls

A list of patients (n = 24) with ESRD who have been undergoing continuous ambulatory peritoneal dialysis (CAPD) or automated peritoneal dialysis (APD) for at least one year was obtained from the Department of Internal Medicine, Division of Nephrology of Istanbul Faculty of Medicine (Istanbul University, Turkey). Patients who had blood transfusions in the last three months or those who experienced any bleeding were excluded. Patients used different dialysis solutions according to their conditions (Baxter Healthcare, www.baxter.com or Fresenius Medical Care AG, www.fresenius.com). 27 individuals were included in the study as controls. Controls were selected for the experiments if (1) Blood urea nitrogen (BUN) levels were within normal range (less than 1.2 mg/dL) and (2) Serum creatinine levels were also within normal range (less than 1.2 mg/dL). Demographic, hematological and biochemical features of two groups are listed in Table 1. This study was approved by the Ethics Committee of Istanbul University, Istanbul Faculty of Medicine (IRB: 956/2015) and all participants submitted written informed consent according to Declaration of Helsinki.

**Table 1:** Clinical and demographic features of the patients and control subjects.

Parameters	Peritoneal Dialysis (n = 24)	Control (n = 27)
**Age (year)**	45 ± 13 (19–61)	39 ± 12 (25–60)
**Sex (M/F)**	12/12	14/13
**Duration of PD (year)**	3.16 ± 4.1 (1–15)	-
**RBC (M/μl)**	3.7 ± 0.6‡	5.3 ± 0.8
**Hb (g/dL)**	11 ± 1.7‡	13.9 ± 0.9
**Hct (%)**	33 ± 5‡	43 ± 3
**MCV (fL)**	90 ± 4.9	88 ± 5.1
**MCH (pg)**	30.1 ± 1.9†	27.8 ± 1.5
**MCHC (g/dL)**	33.8 ± 1.02‡	31.7 ± 1.34
**RDW (%)**	14.2 ± 1.2‡	12.4 ± 7.8
**Blood Urea Nitrogen (BUN) (mg/dL)**	54.09 ± 15.2‡	14.57 ± 4.82
**Serum Creatinine (mg/dL)**	9.66 ± 3.13‡	0.71 ± 0.17
**Uric acid (mg/dL)**	5.37 ± 1.1	4.65 ± 1.73
**Cholesterol (mg/dL)**	207 ± 46	195 ± 29.49
**HDL (mg/dL)**	39.5 ± 11.5	38.8 ± 10.75
**LDL (mg/dL)**	136 ± 42.5	128.63 ± 39.65
**VLDL (mg/dL)**	32.7 ± 11	26.9 ± 18.08
**Triglyceride (mg/dL)**	174.9 ± 78.4	147.11 ± 116.2
**CRP (mg/dL)**	13.5 ± 18.6*	2.76 ± 3.55
**Use of erythropoiesis stimulating agents (use/non use)**	16 / 8	-
**Cause of ESRD**		
**Chronic kidney failure (Etiology unknown)**	12	-
**Polycystic kidney disease**	5	-
**Chronic glomerulonephritis**	2	-
**Nephrosclerosis**	3	-
**Others**	10	-

Difference from control:

*p≤ 0.05,

^†^p ≤ 0.001,

^‡^p≤0.0001, the values are presented as mean ± SD.

### Sample preparation

Fresh venous blood samples were collected from patients and control subjects into 10 ml vacutainer tubes containing K_2_-EDTA. Briefly, whole blood was centrifuged at 1400 x g for 5 min and plasma was separated. For aggregation measurements, erythrocytes were prepared either with autologous plasma or isotonic phosphate buffered saline (PBS, 290 mOsm/kg, pH 7.4) containing 3% Dextran 70 (MW 70 kDa; Sigma Chemical Co., St. Louis, MO, USA) at a hematocrit (Hct) of 45%. For the measurements of erythrocyte deformability, the Hct of whole blood was adjusted to the level of 45%, as well.

### Hematological and biochemical parameters

Hematological parameters of the participants were determined by an electronic hematology analyzer (ABX Micros 60, Montpellier, France). Biochemical parameters of the patients were obtained from the patients records.

#### Viscosity measurements

Whole blood viscosity (BV) at native and corrected Hct (45%) was measured at shear rates of 75, 150, 185, 300, 375, 750, 1350 and 1500 s^-1^ and plasma viscosity was measured at a shear rate of 300 s^-1^. All measurements were performed by a cone/plate viscometer (Wells-Brookfield Cone/Plate Viscometer, Middleboro, MA, USA) at 37 °C.

#### Measurements of erythrocyte deformability and osmotic gradient deformability

Erythrocyte deformability was measured by a laser-assisted optical rotational cell analyzer (LORCA MaxSis, Mechatronics, Netherlands). The methodology that we used in this study was previously described by Hardeman *et al* [14]. Erythrocyte deformability was measured at 37 °C applying ten different shear stresses from 0.3 to 50 Pa and determined by the change in elongation index (EI) with applied shear stresses. Maximal erythrocyte elongation index (EI_max_) and the shear stress required for one-half of this maximal deformation (SS_1*/*2_) were calculated using the Lineweaver-Burke (LB) model [15].

Osmotic deformability of erythrocyte was assessed at 37 °C by the Osmoscan test (LORCA MaxSis, Mechatronics, Netherlands) with a constant shear stress of 30 Pa. Briefly, 200 μl of whole blood was added to 5 ml of PVP solution (Osmolality: 296 mOsm/kg), gently mixed and applied into the measuring chamber. As a measure of deformability, erythrocyte elongation was recorded continuously while the osmolality of the suspending medium was gradually changed from 100 mOsm/kg to 500 mOsm/kg via an automated program in the ektacytometer. The parameters calculated by the device are as follows: maximal elongation index values measured at a shear stress of 30 Pa (EI_max_), minimal elongation index values measured at a shear stress of 30 Pa at low osmolar conditions (EI_min_), measureable elongation index values at a shear stress of 30 Pa at high osmolar conditions (EI_hyper_), osmolality at minimal EI (O_min_), osmolality at maximal EI (O_max_), osmolarity at EI_hyper_ (O_hyper_), and the areas under the individual osmolality-elongation index curves [16].

#### Measurements of erythrocyte aggregation

Erythrocyte aggregation was analyzed by a laser diffraction ektacytometer system (LORCA, MaxSis, Mechatronics, Netherlands). The aggregation process is evaluated by the change in the intensity of back-scattered light by time, and it is plotted as a graph called syllectogram [17]. The following parameters of aggregation were calculated automatically: Extent or amplitude of aggregation (AMP), kinetics of aggregation expressed by the aggregation half-time (t½) and the aggregation index (AI) which shows the kinetics and amplitude of aggregation [17]. The study was performed at 37 °C. Erythrocyte aggregation indices were measured twice in whole blood with their autologous plasma and in suspensions of 3% dextran 70 solution.

#### Assays for oxidative stress and antioxidant status

Enzyme activities of Glutathione peroxidase (GPx), Superoxide dismutase (SOD) and Catalase (CAT) were studied in erythrocyte lysates by assay kits (Cayman Chemical, Michigan, USA). Lipid peroxidation was studied via measuring the amount of Malondialdehyde (MDA) in both erythrocyte and plasma samples by the Lipid Peroxidation Assay kit (ab118970, Abcam, Cambridge, MA, USA) according to the procedures of the manufacturers. All assays were prepared in 96 well plates and studied in duplicates. Measurements were performed spectrophotometrically by a plate reader (Hybrid Reader, Synergy H1, BioTek, VT, USA) and calculations were done by plotting a standard curve except for the GPx assay, which was measured by a time scale.

### Statistical analysis

The results are reported as mean *±* standard deviations. Normality of the data was tested using the Shapiro-Wilks test (GraphPad Software Inc, Release 4.0, USA). For normally distributed dependent variable, parametric test—student’s unpaired *t*-test was used to compare measurements between the two groups. For those parameters that are not normally distributed, nonparametric test—Mann-Whitney U-test was employed for comparisons. Correlation analysis for the results of the enzymatic activity assays with the data from hemorheological studies were performed by Pearson or Spearman tests where applicable. Level of significance was accepted to be 0.05. Non-linear curve fitting to determine EI_max_ and SS_1*/*2_ was carried out using a commercially available software (GraphPad Software Inc, Release 4.0, USA).

## Results

Erythrocyte count, Hct and total hemoglobin concentration were lower in PD patients than that of controls. Red cell distribution width (RDW), mean corpuscular hemoglobin (MCH) and mean corpuscular hemoglobin concentration (MCHC) increased in patients, whereas mean corpuscular volume (MCV) was not significantly different between the patients and controls. Biochemical parameters of the patients are shown in Table 1. The BUN, serum creatinine and C-reactive protein (CRP) levels were significantly higher in PD patients. Patients who had erythropoietin treatment had significantly lower erythrocyte counts (p = 0.028), Hb (p = 0.001) and Hct (p = 0.013) than non-treated patients. MCV, MCH and MCHC values of the patients did not change with erythropoietin usage.

**Fig 1:**
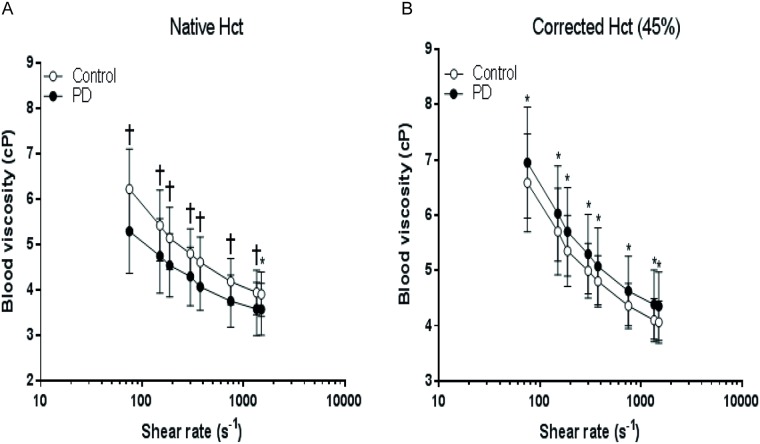
Whole blood viscosity of controls and peritoneal dialysis patients at native (A) and corrected hematocrits (Hct) (B) measured at shear rates between 75 and 1500 s^-1^. Difference from control:*p≤0.05, †p≤0.001, the values are presented as mean ± SD.

The blood viscosity (BV) at native and corrected Hct levels are presented in Fig 1. The BV at native Hct showed significant differences between the two groups at all shear rates (p≤0.05). When Hct was adjusted to 45%, the BV significantly increased in PD patients at all measured shear rates compared to controls (p≤0.05). Furthermore, plasma viscosity was also significantly higher in patients (1.9 ± 0.24 cP) than controls (1.4 ± 0.17 cP, p<0.0001).

**Fig 2:**
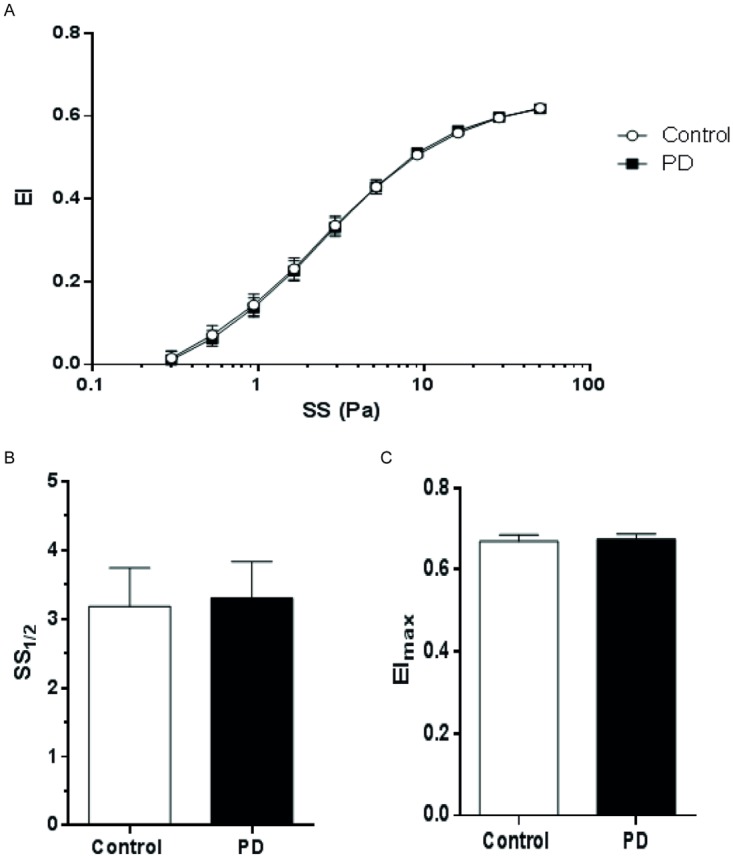
Elongation indexes (EI) of controls and peritoneal dialysis patients measured at shear stresses between 0.3 and 50 Pa. Panel A presents EI—SS relations for controls and peritoneal dialysis patients. Panel B shows the shear stress levels required for half-maximal deformation (SS_1*/*2_), panel C presents maximal elongation index at infinite shear stress (EI_max_). The values are presented as mean ± SD.

Erythrocyte deformability was evaluated through EI, EI_max_ and SS_1*/*2_ values. Maximum elongation index (EI_max_) values were 0.67 ± 0.01 and 0.66 ± 0.01, whereas SS_1*/*2_ values were 3.30 ± 0.52 and 3.18 ± 0.55 in PD patients and controls, respectively (Fig 2B and 2C). Accordingly, erythrocyte deformability was not statistically different in PD patients from controls (Fig 2A).

**Fig 3:**
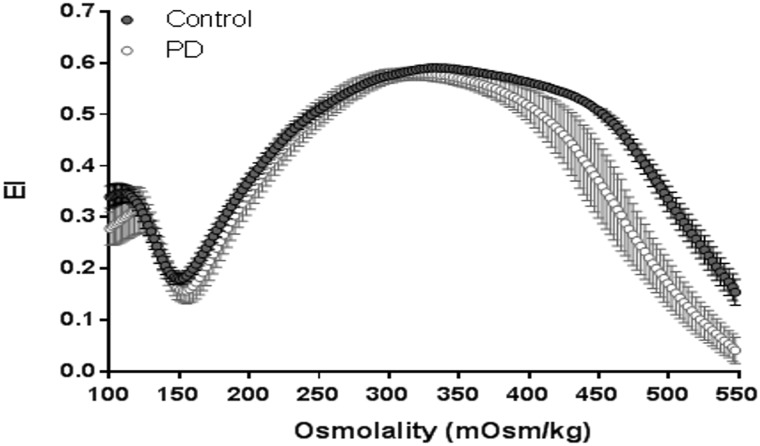
Representative osmotic deformability profiles for control and peritoneal dialysis patients.

Fig 3 presents representative cumulated EI-osmolarity curves. We found significant differences in some parameters of osmotic deformability between patients and controls (Table 2). Minimal elongation index values at low osmolar conditions were significantly lower in PD patients compared to controls (p = 0.042), while maximal elongation index values were similar in both groups (p = 0.12). Measureable elongation index values at high osmolar conditions (EI_hyper_), the osmolarity at minimal EI (O_min_) and the osmolarity at maximal EI (O_max_) were not different in patients from controls (p = 0.11, p = 0.35, p = 0.76 respectively). Furthermore, the osmolarity at EI_hyper_ (O_hyper_) was significantly low in PD patients compared to controls (p = 0.019). The areas under the individual osmolarity-elongation index curves of patients and controls were significantly different (p = 0.012) where patients exhibited lower area values than controls.

**Table 2:** Parameters of osmoscan measurements at shear stress of 30 Pa.

Variable	Peritoneal Dialysis	Control
**Minimal EI**	0.14 ±0.025*	0.17 ± 0.040
**Maximal EI**	0.59 ± 0.011	0.59 ± 0.006
**EI_hyper_**	0.29 ± 0.006	0.30 ± 0.003
**Osmolarity at minimal EI (mOsm/kg)**	157.9 ± 6.32	154.7 ±7.93
**Osmolarity at maximal EI (mOsm/kg)**	332.7 ± 14.59	334.5 ± 14.97
**Osmolarity at EI_hyper_ (mOsm/kg)**	485.9 ± 25.33*	510.5 ± 30.4
**Area (osm-EI curve)**	161.7 ± 7.2*	168.1 ± 4.4

Means ± S.D.

*p ≤0.05, vs. values at 30 Pa.

**Fig 4:**
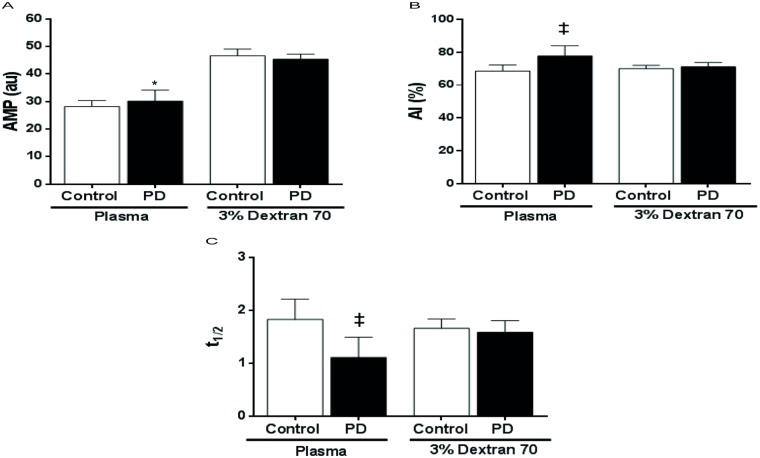
Erythrocyte aggregation changes of controls and peritoneal dialysis patients in plasma and dextran solution. A: Amplitude of aggregation (AMP), B: Aggregation index (AI), C: Aggregation half time (t½). *p ≤0.05, ‡*p* ≤0.0001. The values are presented as mean ± SD.

Erythrocyte aggregation measurements were done both in autologous plasma and 3% Dextran 70. PD patients showed significantly higher AMP and aggregation index (AI) values than controls in plasma (p = 0.034 and p<0.0001, respectively) but not in dextran solution (Fig 4A and 4B). Considering the kinetics of aggregation, PD patients exhibited significantly lower t½ values in plasma (p<0.0001) but not in dextran compared to controls (Fig 4C).

The activities of antioxidant enzymes in patients and controls are shown in Table 3 with their respective units of concentration. GPx and CAT activities in erythrocyte lysates of PD patients were significantly lower than controls (p = 0.0009, p = 0.04, respectively). However, SOD activity increased in erythrocytes of PD patients (p = 0.02). On the other hand, MDA levels in both erythrocyte and plasma samples were higher in patients than controls, which was statistically significant (p = 0.014 and p = 0.01, respectively). Antioxidant enzyme activities were analyzed for any correlation with the hemorheological data. Plasma levels of MDA were positively correlated with t½ values in plasma samples of the patients (r = 0.6, p = 0.003). However, negative correlations were observed with AI (r = -0.6, p = 0.003) and AMP values in plasma (r = -0.6, p = 0.002). Considering erythrocyte deformability, there was no correlation with enzyme activities in patients and controls. Furthermore, activities of GPx, CAT and SOD did not show any correlation with hemorheological parameters.

**Table 3:** The antioxidant enzyme activities and lipid peroxidation biomarker in erythrocyte or plasma samples of PD patients and controls (Average±SD (top), Median; CV (bottom)).

Enzymes (Unit)	Peritoneal Dialysis	Control	p
**GPx (nmol/min/ml)**	62.98 ± 14.95	80.05 ± 15.55	0.0009†
	61.55; 0.23	79.38; 0.19	
**CAT (nmol/min/ml)**	9.62 ± 3.49	11.62 ± 3.32	0.04*
	9.52; 0.36	11.77; 0.28	
**SOD (U/ml)**	151.7 ± 113.1	94.42 ± 44.8	0.02*
	128.1; 0.74	84.79; 0.47	
**MDA in erythrocytes (nmol/μg)**	13.54 ± 0.2	13.25 ± 0.47	0.014*
	13.59; 0.01	13.41; 0.03	
**MDA in plasma (nmol/μl)**	5.04 ± 1.29	4.19 ± 0.91	0.01*
	4.91; 0.25	4.05; 0.21	

SD: Standard deviation, CV: coefficient of variation,

*p ≤0.05,

^†^p≤0.001.

## Discussion

Erythrocytes may be affected from osmotic alterations during peritoneal dialysis, even though they are not exposed to any mechanical contact. It is strongly possible that they are faced with alterations in osmolality more or less during the exchange between blood and peritoneal cavity. In addition, the different dialysis solutions are used with different glucose concentrations for the peritoneal dialysis treatment [18]. Therefore, we measured osmotic deformability that means measuring elongation index as a function of osmolality at a constant shear stress. Osmotic deformability measurements were performed at a constant SS of 30 Pa. Previously, it was shown that the osmotic deformability measurements are the most stable if SS is 30 Pa [19].

EI_min_ represents the point at which the cells were maximally swollen and therefore spherical [16]. We found significantly reduced EI_min_ values in PD patients (Table 2). Low EI_min_ shows us the resistance of the erythrocytes in PD patients decreased due to the hypoosmolality. Whereas, EI_max_ which shows deformability at isotonic level, did not change in patients compared to controls. In addition, we also found significant difference in area measurements between PD patients and controls. Our paper presents the first study of the osmotic deformability changes of erythrocytes of the peritoneal dialysis patients with ESRD. The measurements of osmotic deformability usually detect minor alterations in erythrocyte surface area or volume, and in intracellular viscosity resulting from changes in MCHC [16]. Reduced surface area of cells shows an increase in EI_min_ despite their normal water contents, because less swelling is required to expand their reduced surface area. Therefore, any changes in osmotic deformability should be considered as an important sign for cellular defects and it might provide information about the level of damage in erythrocytes. Although there was no significant change in erythrocyte deformability, osmotic gradient deformability significantly decreased in the EI_min_ and O_hyper_ parameters. It has been shown that decrement in EI_min_ and O_hyper_ occurs as a result of increase in intracellular viscosity which results from osmotic water loss and Hb changes [20]. The significant rise in MCH and MCHC of our patients supports previous findings and explains our osmotic deformability decrements. Similar MCV values in PD patients and controls revealed that there was not any change in erythrocyte volume, so intracellular viscosity alterations are substantially responsible for the osmotic deformability loss.

Anemia is a common problem for patients suffering from ESRD due to the insufficiency of erythropoietin production. Previously, it was shown that erythrocyte survival rate of these patients was less than approximately 20% of controls in both hemodialysis (HD) and peritoneal dialysis (PD) patients and most of ESRD patients were supported by an erythropoiesis stimulating agent (ESA) [6]. In our study, all patients had lower Hct than control subjects. In fact, high Hct and Hb were not desirable for PD treatment and ESA-treatment was only applied if the Hct was too low which causes oxygen insufficiency [21–24]. On the other hand, Dortmandy *et al*. supposed erythropoietin therapy might render the cells less deformable and thus more likely to obstruct small vessels [25]. But later on, it was shown that erythropoietin treatment did not change erythrocyte deformability but it increased the whole blood platelet aggregation [23]. These findings let us to ignore the ESA-treatment for the erythrocyte hemorheological measurements.

In the circulation, blood viscosity varies with shear rate conditions: blood becomes less viscous at high shear rates. At the low shear rates, erythrocytes normally start to aggregate and the erythrocyte aggregation is disrupted at high shear rates. However, non-physiological conditions, such as acute effects of inflammation (increased levels of fibrinogen and other large serum proteins) cause larger aggregates in the vascular network. So, the large aggregates may lead to high blood viscosity causing slow flow stream in the vasculature [26]. According to our data, patients have high CRP, creatinine and BUN levels and it is expected for the erythrocyte to be affected by these circumstances. We measured erythrocyte aggregation in PD patients. Although in the literature there are many hemorheological measurements in hemodialysis patients including erythrocyte aggregation, studies for the evaluation of hemorheological parameters in peritoneal dialysis are limited [27]. The possible reasons might be that the aggregation behavior of erythrocyte has not yet been widely put into practice and the difficulties of following the variable procedures in peritoneal dialysis patients. Recently, Fontana *et al*. showed that the erythrocyte aggregation was not significantly different in PD patients compared to control subjects [27].

However, RBC aggregation was measured indirectly in their study [17], whereas RBC aggregation is presented here through direct parameters; AI, AMP and t_1/2_. We showed that the AI was significantly higher in patient plasma (Fig 4). Our result is in contrast with the previous study [27]. On the other hand, another study showed that the patients with renal insufficiency had significantly increased AI levels compared to the volunteers [13]. In our study, we also presented two different parameters: t_½_ as kinetics of aggregation half time and AMP which indicates total extent of aggregation. The AMP was not significantly different in PD patients compared to the controls in dextran, whereas it was significantly increased in PD patients in plasma. We interpreted that increased AMP is more relevant for plasma rather than erythrocyte itself. However, according to decreased t_½_ in plasma, formation of rouleaux is faster in plasma than in dextran solution for PD patients compared to the controls. Hence, we concluded plasma factors (uremia, CRP, globulins etc.) cause rouleaux formation to occur faster than it does in the control group, which is a risk factor for microcirculation. These aggregation dynamics are also presented for the first time for PD patients.

Previously, it was shown that PD patients have higher plasma viscosity than the HD patients and control subjects [28]. McGregor *et al* demonstrated that higher plasma viscosity in PD patients comparing to HD patients that was mainly due to higher fibrinogen levels [29]. More recently, another study confirmed that the plasma viscosity levels are high in PD patients, but they explained it via high γ-globulin values [27]. In our study we also found higher plasma viscosity levels in PD patients than in control. Similarly, we found significantly higher BV values in patients than in control when Hct was corrected to 45% [27, 29], while the BV at their native Hct was significantly lower in PD patients compared to controls. Viscosity is an important parameter for the cardiovascular system and high viscosity has been accepted as a risk factor for a long time. Our results show that, in PD patients, Hct should be less than normal for better microcirculation and macrocirculation.

Previous studies showed that the antioxidant capacities of blood was altered in chronic renal failure and dialysis patients [12, 30–32]. In this study, the antioxidant capacity of PD patients was worsened significantly with a decrease in GPx and CAT and with an increase in MDA (Table 3), confirming previous studies [8, 30]. The higher MDA levels imply an increase in production of oxygen derived free radicals [33]. It is possible that elevated MDA levels are due to decreased antioxidant activity. However, we found higher SOD activity in PD patients than controls, which is also showed by Martin-Mateo *et al* [31]. Moreover, in the same study, they found high CAT activity in contrast to our result. Increased SOD activity in PD patients in our study could be due to the altered concentrations of essential trace elements like copper (Cu) which is an important co-factor of SOD enzyme. Since Cu concentration is known to be increased in these patients due to the dialysis [34], it is possible that SOD activity is elevated in PD patients in our study. On the other hand, we showed that these alterations did not significantly affect erythrocyte deformability because none of the enzyme activities showed any correlation with deformability.

In conclusion, our study presents clear evidence that peritoneal dialysis is associated with erythrocyte rheological disturbances expressed by increased erythrocyte aggregation and the alteration of total aggregation extent, decreased osmotic deformability, increment in plasma viscosity and blood viscosity. In PD patients, imbalance between oxidant/antioxidant status accompanies these erythrocyte rheological distortions, so such alterations together in erythrocyte would increase *in vivo* destruction and reduce their life span, contributing to anemia. This study also showed that an osmotic gradient ektacytometer is a very sensitive tool for detecting minor erythrocyte damages in PD.

## References

[1] Lang KS, Lang PA, Bauer C, Duranton C, Wieder T, Huber SM, et al Mechanisms of suicidal erythrocyte death. Cell Physiol Biochem. 2005;15(5):195–202. 10.1159/000086406 15956782

[2] Lang KS, Myssina S, Lang PA, Tanneur V, Kempe DS, Mack AF, et al Inhibition of erythrocyte phosphatidylserine exposure by urea and Cl. Am J Physiol Renal Physiol. 2004;286(6):F1046–53. 10.1152/ajprenal.00263.2003 15130896

[3] Lang KS, Duranton C, Poehlmann H, Myssina S, Bauer C, Lang F, et al Cation channels trigger apoptotic death of erythrocytes. Cell Death Differ. 2003;10(2):249–56. 10.1038/sj.cdd.4401144 12700653

[4] Lang PA, Kaiser S, Myssina S, Wieder T, Lang F, Huber SM. Role of Ca2+-activated K+ channels in human erythrocyte apoptosis. Am J Physiol Cell Physiol. 2003;285(6):C1553–60. 10.1152/ajpcell.00186.2003 14600080

[5] Sotirakopoulos N, Tsitsios T, Stambolidou M, Athanasiou G, Peiou M, Kokkinou V, et al The red blood cell deformability in patients suffering from end stage renal failure on hemodialysis or continuous ambulatory peritoneal dialysis. Ren Fail. 2004;26(2):179–83. 15287203 10.1081/jdi-120038517

[6] Vos FE, Schollum JB, Coulter CV, Doyle TC, Duffull SB, Walker RJ. Red blood cell survival in long-term dialysis patients. Am J Kidney Dis. 2011;58(4):591–8. 10.1053/j.ajkd.2011.03.031 21715072

[7] Ly J, Marticorena R, Donnelly S. Red blood cell survival in chronic renal failure. Am J Kidney Dis. 2004;44(4):715–9. 15384023

[8] Stepniewska J, Dolegowska B, Popinska M, Salata D, Budkowska M, Golembiewska E, et al Prooxidative-antioxidative balance of cells in different types of renal replacement therapy. Blood Purif. 2014;37(1):4–11. 10.1159/000356806 24481175

[9] Puchades MJ, Saez G, Munoz MC, Gonzalez M, Torregrosa I, Juan I, et al Study of oxidative stress in patients with advanced renal disease and undergoing either hemodialysis or peritoneal dialysis. Clin Nephrol. 2013;80(3):177–86. 10.5414/CN107639 23782545

[10] Nowak-Piszczek E, Wyrwicz G, Dabrowski Z, Smolenski O, Spodaryk K. Deformability and enzymes activities of red blood cells in hemodialysed and peritoneal dialysed patients with chronic renal disease. Clin Hemorheol Microcirc. 2003;28(4):251–7. 12897415

[11] Ibrahim FF, Ghannam MM, Ali FM. Effect of dialysis on erythrocyte membrane of chronically hemodialyzed patients. Ren Fail. 2002;24(6):779–90. 12472200 10.1081/jdi-120015680

[12] Szikszai Z, Ujhelyi L, Imre SG. Effect of hemodialysis on the deformability and lipid peroxidation of erythrocytes in chronic renal failure. Clin Hemorheol Microcirc. 2003;28(4):201–7. 12897411

[13] Pietrzycka A, Sloczyńska K, Teleglȯw A, Marchewka A, Drozdz M, Stêpniewski M, et al Aggregation and elongation indices of erythrocytes of patients with chronic renal insufficiency relate to antioxidant capacity FRAP and CAT/SOD ratio values. Nephrol Dial Pol. 2010;14:22–6.

[14] Hardeman MR, Goedhart PT, Schut NH. Laser-assisted optical rotational cell analyser (L.O.R.C.A.); II. Red blood cell deformability: Elongation index versus cell transit time. Clin Hemorheol Microcirc. 1994;14:619–30.

[15] Baskurt OK, Hardeman MR, Uyuklu M, Ulker P, Cengiz M, Nemeth N, et al Parameterization of red blood cell elongation index—shear stress curves obtained by ektacytometry. Scand J Clin Lab Invest. 2009;69(7):777–88. 10.3109/00365510903266069 19929721

[16] Clark MR, Mohandas N, Shohet SB. Osmotic gradient ektacytometry: comprehensive characterization of red cell volume and surface maintenance. Blood. 1983;61(5):899–910. 6831052

[17] Hardeman MR, Dobbe JG, Ince C. The Laser-assisted Optical Rotational Cell Analyzer (LORCA) as red blood cell aggregometer. Clin Hemorheol Microcirc. 2001;25(1):1–11. 11790865

[18] Heimbürger O, Blake PG. Apparatus for Peritoneal dialysis In: Daugirdas JT B P, Ing TS, editors. Handbook of Dialysis. Philadelphia: Lippincott Williams and Wilkins; 2015 pp. 408–24.

[19] Nemeth N, Kiss F, Miszti-Blasius K. Interpretation of osmotic gradient ektacytometry (osmoscan) data: a comparative study for methodological standards. Scand J Clin Lab Invest. 2015;75(3):213–22. 10.3109/00365513.2014.993695 25594795

[20] Mohandas N, Clark MR, Jacobs MS, Shohet SB. Analysis of factors regulating erythrocyte deformability. J Clin Invest. 1980;66(3):563–73. 10.1172/JCI109888 6156955 PMC371685

[21] Casati S, Campise M, Crepaldi M, Lobo J, Graziani G, Ponticelli C. Haemodialysis efficiency after long-term treatment with recombinant human erythropoietin. Nephrol Dial Transplant. 1989;4(8):718–20. 2510080 10.1093/ndt/4.8.718

[22] Casati S, Passerini P, Campise MR, Graziani G, Cesana B, Perisic M, et al Benefits and risks of protracted treatment with human recombinant erythropoietin in patients having haemodialysis. Br Med J (Clin Res Ed). 1987;295(6605):1017–20. 10.1136/bmj.295.6605.1017 PMC1248067 3120854

[23] Taylor JE, MacTier RA, Henderson IS, Belch JJ, Stewart WK. Dialysis efficiency in continuous ambulatory peritoneal dialysis patients treated with erythropoietin. Perit Dial Int. 1992;12(2):221–6. 1586685

[24] Borzych-Duzalka D, Bilginer Y, Ha IS, Bak M, Rees L, Cano F, et al Management of anemia in children receiving chronic peritoneal dialysis. J Am Soc Nephrol. 2013;24(4):665–76. 10.1681/ASN.2012050433 23471197 PMC3609132

[25] Dormandy JА, Flute Р, Маtrai А, Вogar L, Мikita J. The new St. George's blood filtrometer. Clin Hemorheol. 1985;5(6):975–83.

[26] Piagnerelli M, Boudjeltia KZ, Vanhaeverbeek M, Vincent JL. Red blood cell rheology in sepsis. Intensive Care Med. 2003;29(7):1052–61. 10.1007/s00134-003-1783-2 12802488

[27] Fontana F, Ballestri M, Makomi C, Morandi R, Cappelli G. Hemorheologic alterations in peritoneal dialysis. Clin Hemorheol Microcirc. Forthcoming 2016. 10.3233/CH-16152 27340762

[28] Feriani M, Kimmel PL, Kurantsin-Mills J, Bosch JP. Effect of renal replacement therapy on viscosity in end-stage renal disease patients. Am J Kidney Dis. 1992;19(2):131–9. 1739094 10.1016/s0272-6386(12)70121-x

[29] McGregor D, Shand B, Lynn K. A controlled trial of the effect of folate supplements on homocysteine, lipids and hemorheology in end-stage renal disease. Nephron. 2000;85(3):215–20. doi: 45664 10867536 10.1159/000045664

[30] Clermont G, Lecour S, Lahet J, Siohan P, Vergely C, Chevet D, et al Alteration in plasma antioxidant capacities in chronic renal failure and hemodialysis patients: a possible explanation for the increased cardiovascular risk in these patients. Cardiovasc Res. 2000;47(3):618–23. 10963735 10.1016/s0008-6363(00)00117-6

[31] Martin-Mateo MC, del Canto-Jafiez E, Barrero-Martinez MJ. Oxidative stress and enzyme activity in ambulatory renal patients undergoing continuous peritoneal dialysis. Ren Fail. 1998;20(1):117–24. 9509565 10.3109/08860229809045094

[32] Al-Hashimi AF, Mohammed FH, Al-Khazragi AS. Oxidative stress in chronic renal failure patients treated by peritoneal dialysis. Saudi Med J. 2004;25(9):1186–92. 15448763

[33] McGrath LT, Douglas AF, McClean E, Brown JH, Doherty CC, Johnston GD, et al Oxidative stress and erythrocyte membrane fluidity in patients undergoing regular dialysis. Clin Chim Acta. 1995;235(2):179–88. 7554272 10.1016/0009-8981(95)06027-x

[34] Guo CH, Wang CL, Chen PC, Yang TC. Linkage of some trace elements, peripheral blood lymphocytes, inflammation, and oxidative stress in patients undergoing either hemodialysis or peritoneal dialysis. Perit Dial Int. 2011;31(5):583–91. 10.3747/pdi.2009.00225 20592101

